# Computer-aided detection to strengthen tuberculosis screening and diagnosis in Pakistan

**DOI:** 10.1097/MS9.0000000000003922

**Published:** 2025-09-17

**Authors:** Hamza Sajid, Hamdia Gul Aslam, Noor un nisa Irshad, Sakan Binte Imran

**Affiliations:** a Department of Medicine, Allama Iqbal Medical College, Lahore, Pakistan; b Department of Medicine, Jinnah Sindh Medical University, Karachi, Pakistan

*Dear Editor,*

Tuberculosis (TB) is one of the biggest health threats globally and remains a major concern in Pakistan, a country among the top five high-burden TB nations globally. The estimated annual number of new TB cases in Pakistan is approximately 623 000. Another recent estimate is a figure closer to 686 000 new cases in 2023, with an incidence rate of around 277 per 100 000 population^[1]^. Despite the efforts to control TB, Pakistan still faces a considerable diagnostic gap. The capacity to accurately screen and diagnose TB remains severely limited throughout the country^[1]^.

Traditional diagnostic techniques include X-rays and sputum analysis tests, and GeneXpert MTB/RIF assay, but they all face implementation challenges in Pakistan. Sputum smear microscopy is one of the methods widely used in public health programs, but it offers low sensitivity, especially among children^[2]^. The GeneXpert MTB/RIF assay offers greater accuracy, but the high cost and limited infrastructure access set it back^[3]^. Furthermore, the use and interpretation of chest radiography are restricted in rural areas due to a lack of qualified radiologists. There is an urgent need for screening methods that are accurate, scalable, and quick. To cover this diagnostic gap, computer-aided detection (CAD) will be a savior for Pakistan. A comparative summary of major TB screening modalities is provided in Table 1.

**Table 1:** Comparison of tuberculosis screening modalities in resource-limited settings

Feature	Sputum smear microscopy	GeneXpert MTB/RIF	Chest radiography (human reader)	CAD
Sensitivity	Moderate; misses paucibacillary disease and is reduced in children/HIV-positive populations^[1]^.	High diagnostic accuracy for pulmonary TB and rapid rifampicin resistance detection; substantially more sensitive than smear^[2]^.	Variable and operator-dependent; performance influenced by reader expertise and workload.	Meets WHO triage targets at fixed thresholds in multi-site evaluations; competitive with or exceeding human readers in several settings^[3]^.
Cost (per screened person)	Low	Higher (cartridge-based, device costs)	Moderate (equipment + staff time)	Lower than radiologist reading at scale; favorable national-scale cost projections in Pakistan^[4]^.
Infrastructure needs	Basic microscopy lab	Instrument platform, stable power, cartridge supply chain	Radiography suite (digital/analog)	Digital X-ray + computer; compatible with mobile units; offline/low-connectivity options available^[3]^.
Scalability/throughput	Constrained by manual workload and lab capacity	Constrained by instrument capacity and cartridge availability	Limited by the availability of trained readers	High throughput; automated reads enable large-volume triage and reduced confirmatory testing burden^[3]^.
Human resource requirement	Trained lab technicians	Trained lab staff; platform operation & QA	Radiologist or trained reader essential	Minimal specialist interpretation needed; radiographer-led workflows feasible^[3]^.

TB, tuberculosis; HIV, human immunodeficiency virus; CAD, computer-aided detection; WHO, World Health Organization.

This table summarizes key features of major tuberculosis diagnostic tools, comparing sensitivity, cost, infrastructure, scalability, and human resource needs.

CAD refers to the use of artificial intelligence (AI) algorithms to interpret medical images. CAD systems such as CAD4TB, qXR, and Lunit INSIGHT CXR use deep learning algorithms to automatically analyze digital chest X-rays and use heatmaps or scores to identify potential TB indications. Healthcare professionals can quickly identify patients who may need confirmatory testing, like GeneXpert, which provides an anomaly score or heatmap^[4–6]^.

In environments with limited resources, these technologies provide increased consistency and efficiency^[7]^. Recent studies further underscore the impactful role of CAD tools in enhancing TB screening. In one study, individuals with diabetes exhibited significantly higher CAD TB abnormality scores and a greater prevalence of cavitary lesions outside upper lung zones, suggesting that CAD can sensitively detect more extensive disease presentations in high-risk subgroups^[8]^. Cost analyses from Pakistan confirmed that CAD screening is substantially cheaper than radiologist interpretation, making it suitable for large-scale use^[9]^. Field evaluations in Peshawar also demonstrated high sensitivity (83.2%) at practical thresholds, reinforcing CAD as a feasible triage tool in resource-limited settings^[10]^.

Pakistan should incorporate CAD along with radiography in tertiary care hospitals in areas where there is a huge load of TB and train hospital staff accordingly. Mobile screening vans and camps should be set up in far-off areas where people have no easy access to hospitals. The government should seek help from NGOs and other private working firms to raise awareness about TB and to encourage people to screen and seek treatment. Contracts should be made with private companies and the WHO in order to ensure the cost-effective implementation of CAD technology all over Pakistan. CAD, along with chest radiography, has been implemented in some areas of Pakistan, like Karachi, and it has given promising results in rapidly screening and diagnosing people. The Government of Pakistan should take steps to implement the use of CAD for TB all over the country, especially in low-resource settings.

Besides its benefits, several limitations warrant consideration. CAD performance may vary with population characteristics and comorbidities such as HIV or diabetes^[7]^. Moreover, the cost of CAD, false positive results, and ethical challenges should be considered. In conclusion, CAD integration into traditional techniques can help Pakistan bridge its persistent gap in screening and diagnosis of TB. Further research is needed in countries like Pakistan to generate evidence for its worldwide implementation, cost-effectiveness, and limitations. With the government’s effective planning, public awareness, and support from global health organizations, CAD could play a transformative role in accelerating Pakistan’s progress toward eradicating this disease from the country.

## Data Availability

Not applicable.
